# Supplementary figures and images for: Transcriptome differential expression analysis of defoliation of two different lemon varieties
Source: PeerJ. 2024 Apr 26;12:e17218. doi: 10.7717/peerj.17218 (PMC11057431; doi:10.7717/peerj.17218)

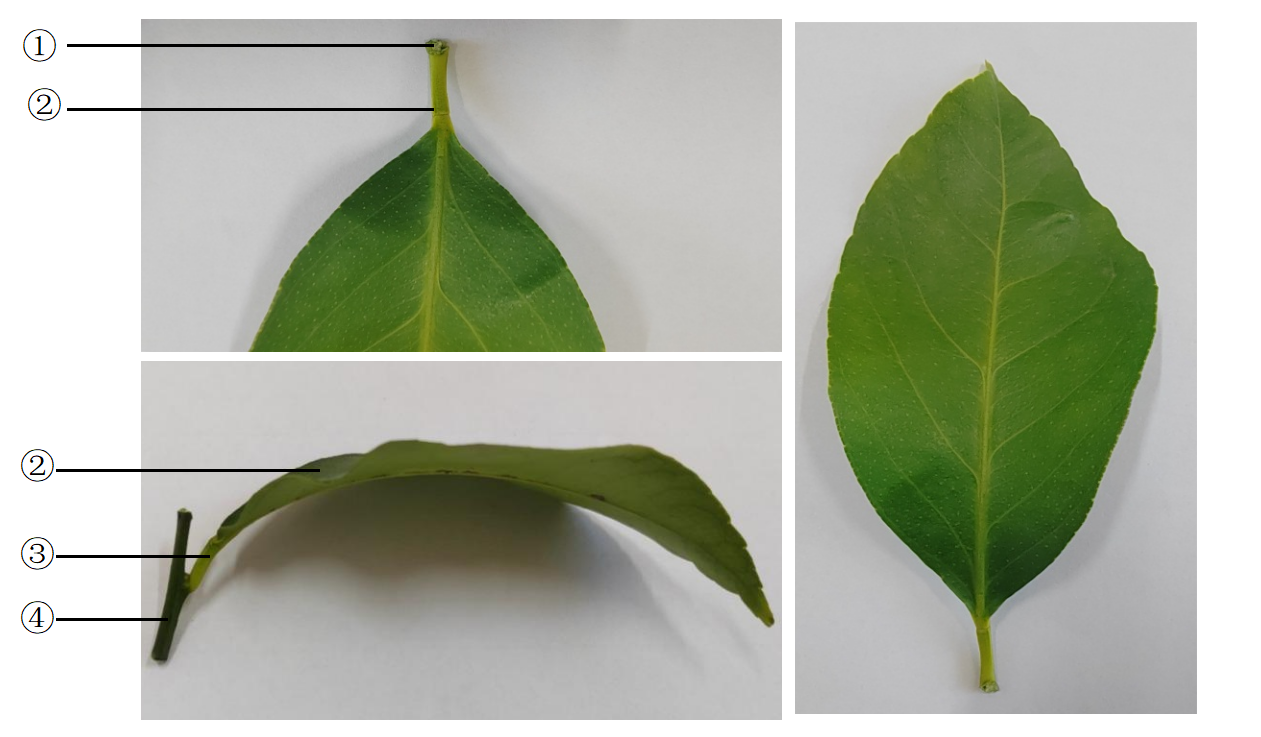

Supplement: Supplemental Information 1 — ①: Abscission zone parts of petioles(Sampling site is 0.3 to 0.5 cm from the base); ②: blade (of propellor); ③: petioles; ④: Petiole-bearing branchlets. [file peerj-12-17218-s001.png]

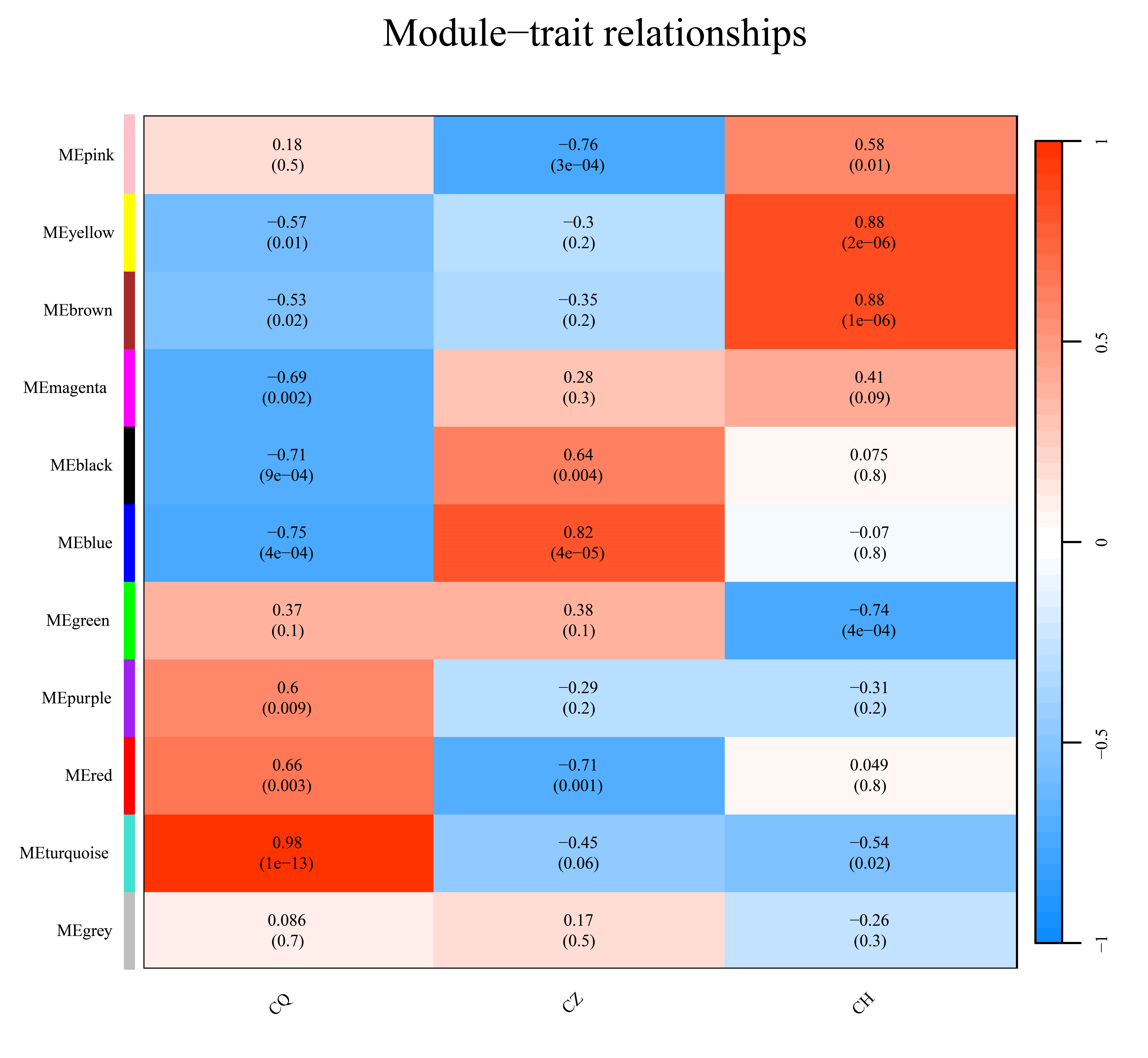

Supplement: Supplemental Information 2 [file peerj-12-17218-s002.png]

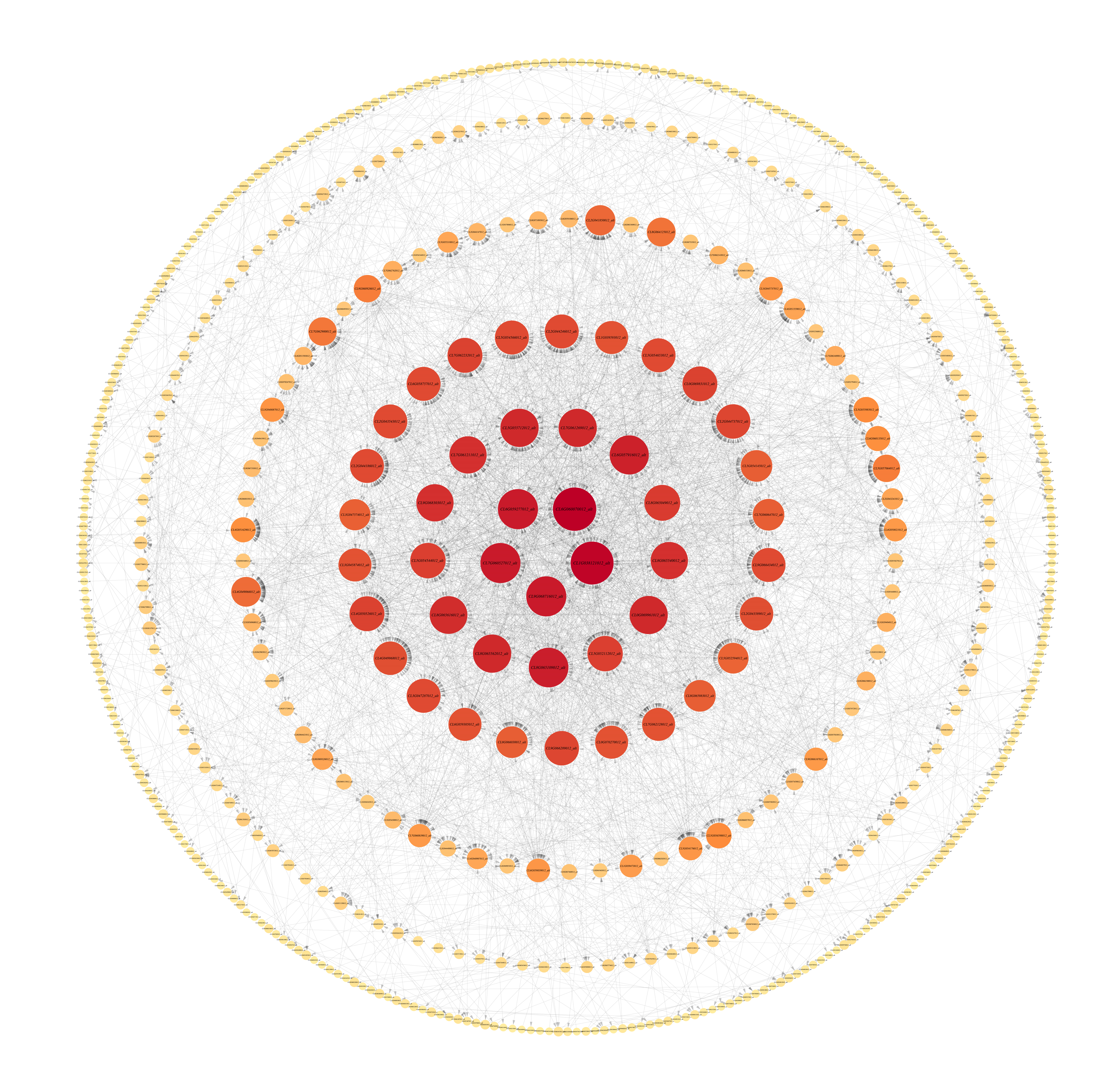

Supplement: Supplemental Information 3 [file peerj-12-17218-s003.png]
